# 

**Published:** 1890-02-15

**Authors:** 


### NEW OFFICES IN THE STRAND.

The Hospital *is now published at* **140, Strand, W.C.** *It has been found desirable to establish* The Hospital *in this central and convenient locality, owing to the rapid extension of the business of this paper of late.*

### NOTICE TO CORRESPONDENTS.

All MS., Letters, Books for Review, and other matters intended for the Editor should be addressed The Editor, The Lodge, Porchester Square, London, W.

**Notice to Advertisers and Subscribers.**

All Advertisements, Orders for Copies of The Hospital, and Business Communications should be addressed to The Publisher, *not to the Editor*, The Hospital, 140, Strand, W.C.

**Bound Volumes of "The Hospital."**

Vol. VI., containing full page portraits of T.R.H. the Prince and Princess of Wales, and Miss Florence Nightingale, is now ready, 4s. post free. Cases for binding may be had of the Publisher, at 1s. 6d. each.

**To Residents, Secretaries, and Matrons.**

The Editor will be glad to have the Regulations and each Report as issued by Asylums, Hospitals, Convalescent and Nursing Institutions, as well as those of other Charities, and an early copy in duplicate of all Appeals and Festival Notices, etc., addressed to *The Editor, The Lodge, Porchester Square, London, W.* Any superintendent, secretary, matron, or other chief official who regularly sends such information may be registered, on application to the Publisher, to receive free of cost a cover for binding The Hospital in half-yearly volumes.

### Scraps and Gleanings.

Peterborough Infirmary is £300 in debt.

Nantwich Workhouse Hospital is to be enlarged at a cost of £5,000.

It is proposed to make the Johnson Hospital, Spalding, a free institution.

Tetbury Cottage Hospital treated 38 patients last year. Expenditure £194.

Miss F. M. Dickinson has been appointed house-surgeon to Belgrave Hospital for Children.

Harrogate Cottage Hospital has cleared off the debt of £70 with which it opened last July.

The third centenary of the invention of the microscope will be celebrated this year at Antwerp.

Cardiff Union Hospital is to have a new wing and a house surgeon. This is a great move in the right direction.

The *Times* has the audacity to state that the sacred Ganges has become nothing more nor less than a common sewer.

The Lord Mayor will preside at the public dinner in aid of University College Hospital on February 25th.

The Royal Albert Asylum, Lancaster, has received £200 from the executors of the late Mr. James Cook, of Stretford.

Mr. Keith D. Young read a paper on "Dwellings for the Labouring Classes" at the Sanitary Institute on Wednesday last.

The medical staff of Dewsbury Infirmary have been presented with articles to the value of £200 in recognition of their services.

There was a crowded attendance at the annual meeting of Pendleton Provident Dispensary. The dispensary continues to flourish.

Worthing Infirmary has decided to have four medical officers instead of three. There seems to be no reason for this increase of the staff.

Doctor Richard Douglas Powell has been appointed Physician-in-Ordinary to the Queen, in succession to the late Sir William Gull.

Baron Tenval, of Nice, offers a prize of £120 for the best portable apparatus to improve the hearing of persons suffering from partial deafness.

Lord George Hamilton will take the chair at the annual court of the Seamen's Hospital Society at the Hotel Metropole at three o'clock on February 18th.

Mr. J. Courtenay Lord, Chairman of the Birmingham Orthopædic Hospital, has been presented by his colleagues on the committee with an illuminated address.

It is reported from St. Petersburg that the Russian physician, Dr. Bapchinski, announces that he has discovered a cure for diphtheria. He says the disease is easily curable by inoculation of erysipelas.

A course of lectures for Sanitary officers commences at the Sanitary Institute on February 18th. Fee for the course of thirteen lectures 6s. Amongst the lecturers will be Sir Douglas Galton and Dr. Louis Parkes.

The Children's Hospital in Great Ormond Street has received a legacy of £60,000, and of £5,000 for the buiding fund. The contract for the erection of the new wing has just been signed, and the work will commence at once.

A few years ago there were nine physician baronets. We have now only Sir William Jenner and Sir Andrew Clark. The *Medical Press and Circular* invites Her Majesty's attention to this painful dearth of baronet-doctors.

The influenza is increasing in the city of Mexico, and so many deaths have occurred that there are not enough hearses to convey the bodies. President Diaz and the members of the Cabinet have contributed to assist destitute sufferers from the malady.

The annual meeting of the supporters of Henshaw's Blind Asylum was held in Manchester, Mr. William Armitage in the chair. The report stated that during the 50 years' existence of the charity about 1,000 blind persons had enjoyed its fostering care.

The National Health Society are arranging with lady teachers to give instructions to shop girls, the members of the Girls' Friendly Society, and other young women on "Household Management," "How to have a Healthy Home." "A Working Woman's Day," etc.

The Warneford Hospital ball took place on the 22nd ult., at the Town Hall, Leamington. The committee had the good fortune this year to secure the Countess of Warwick as the lady patroness, and the lists of patrons and patronesses included the high sheriff of the county.

The most remarkable fact in the Registrar-General's quarterly return is the extraordinarily low mortality from small-pox, which only amounted to 28 in the three months ending December, the smallest number of recorded deaths in any previous year being 275 in 1886.

When the men employed in Portsmouth Dockyard should have gone to the pay-table last week it was found that 600 were absent, chiefly owing to influenza. Sickness among the sailors, however, is decreasing, and 120 men were discharged from Haslar Hospital during one day.

At the Manchester County Police-court, Strangeways, Mr. J. H. P. Leresche, the stipendiary magistrate. presented certificates for efficiency in ambulance work to thirty members of the county force who have been successful in the examinations of the St. John Ambulance Association.

The fifty-ninth session of the Army Medical School was concluded on the 3rd inst., and the prizes distributed at the Royal Victoria Hospital, Netley, in the presence of the professors of the school, the principal medical officers of the hospital, Sir Joseph Fayrer, K.C.S.I., and other visitors.

At the annual meeting of the City of London Truss Society, Mr. John Norbury presided, it was reported that the total income of the society last year was £4,685, as against £6,042 in the previous year. During the year 10,065 patients of both sexes were relieved, being 543 more than in the previous year.

A large deputation attended the meeting of the Fulham Vestry for the purpose of requesting the members to oppose the proposal of the Metropolitan Asylums Board to purchase three acres of additional land in order to enlarge the Western District Fever Hospital at Fulham. The vestry agreed to oppose the scheme.

